# The pleiotropic roles of cGAS–STING signaling in the tumor microenvironment

**DOI:** 10.1093/jmcb/mjac019

**Published:** 2022-03-23

**Authors:** Jun Li, Samuel F Bakhoum

**Affiliations:** Human Oncology and Pathogenesis Program, Memorial Sloan Kettering Cancer Center, New York, NY 10065, USA; Department of Radiation Oncology, Memorial Sloan Kettering Cancer Center, New York, NY 10065, USA; Human Oncology and Pathogenesis Program, Memorial Sloan Kettering Cancer Center, New York, NY 10065, USA; Department of Radiation Oncology, Memorial Sloan Kettering Cancer Center, New York, NY 10065, USA

**Keywords:** immunity, the tumor microenvironment, DNA sensing

## Abstract

Cytosolic DNA is prevalent in cells constituting the tumor microenvironment (TME) and can activate the cyclic guanosine monophosphate–adenosine monophosphate synthase (cGAS)–stimulator of interferon genes (STING) innate immune pathway. The initiation, transmission, and execution of the cGAS–STING pathway can take place among different cell types within the TME and thus cGAS–STING may play opposing roles in driving tumor progression in addition to its tumor cell-intrinsic role. Herein, we review recent advances in the cGAS–STING field with a focus on its crosstalk with other signaling pathways in the TME. Future efforts to depict a more detailed picture of the roles of cGAS–STING signaling in the TME will help design a better cancer treatment regime by targeting the cGAS–STING pathway more precisely.

## Introduction

As the major carrier of genetic information in cells, DNA is compartmentalized by membrane-enclosed structures, including primary nuclei, mitochondria, and chloroplasts. However, during viral or intracellular bacterial infections, DNA species become exposed to the cytosol and act as pathogen-associated molecular pattern molecules to trigger innate immune responses. One of the key downstream signaling pathways is type I interferon (IFN), wherein a broad range of antimicrobial genes, known as interferon-stimulated genes (ISGs), is upregulated in an interferon regulatory factor 3 (IRF3)- and nuclear factor kappa B (NF-κB)-dependent manner (Ishii et al., 2006; Stetson and Medzhitov, 2006).

To identify proteins that function upstream of the type I IFN response, several groups independently identified an endoplasmic reticulum (ER)-resident protein, stimulator of interferon genes (STING; also known as ERIS, MITA, and MPYS), whose overexpression induces ISG expression in 293T cells (Ishikawa and Barber, 2008; Zhong et al., 2008; Sun et al., 2009). Subsequent investigation illuminated that STING is especially required for the viral cytosolic dsDNA-mediated IFN response and its loss severely compromises the ability of host cells to resist viral infection (Ishikawa et al., 2009).

**Figure 1:**
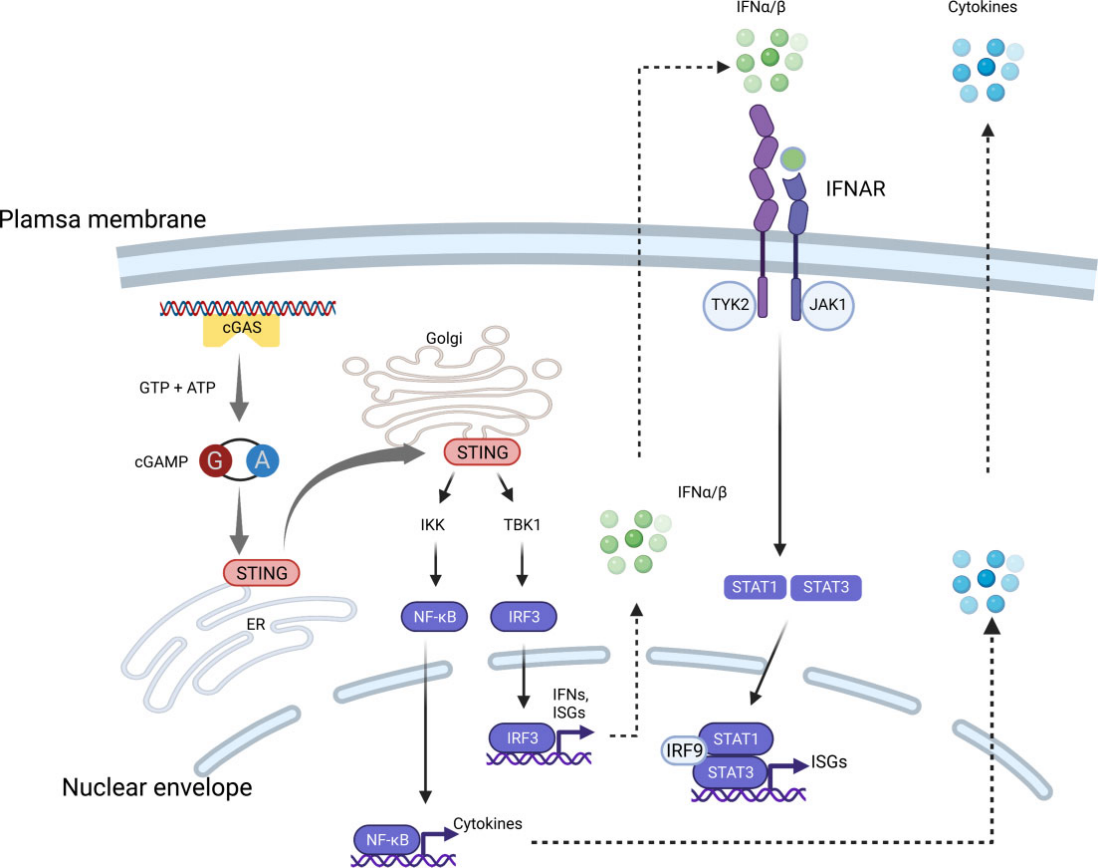
The cGAS–STING pathway senses cytoplasmic DNA. DNA-bound cGAS synthesizes cGAMP, which activates the ER-resident STING. Activated STING translocates to the Golgi and activates IRF3 and NF-κB transcription programs to synthesize IFNs, ISGs, and cytokines. IFNs can be recognized by IFNAR to stimulate STAT1/3 signaling. Created with BioRender.com.

The molecular mechanism by which dsDNA activates STING was further unveiled by the identification of cyclic guanosine monophosphate (GMP)–adenosine monophosphate (AMP) synthase (cGAS; also known as MB21D1) as the direct cytosolic dsDNA sensor (Sun et al., 2013). Binding by dsDNA triggers a conformational change and activates cGAS, which subsequently converts adenosine triphosphate and guanosine triphosphate into 2′3′-cyclic GMP–AMP (cGAMP). cGAMP is the natural ligand of STING, and cGAMP-bound STING polymerizes and translocates from the ER to the Golgi apparatus via an ER–Golgi intermediate compartment (Ishikawa et al., 2009; Saitoh et al., 2009; Civril et al., 2013; Gao et al., 2013; Li et al., 2013; Wu et al., 2013; Zhang et al., 2014; Dobbs et al., 2015; Zhou et al., 2018). Activated STING serves as a platform for IRF3 phosphorylation catalyzed by TANK-binding kinase 1 (TBK1). Phosphorylated IRF3 then dimerizes, translocates to the nucleus, and activates the transcription of ISGs. STING can also activate IκB kinase (IKK) (Ishikawa et al., 2009). IKK then phosphorylates and inactivates the inhibitors of NF-κB. Activated NF-κB in turn stimulates the expression of IFNs and a host of inflammatory cytokines (Figure 1).

The early preclinical studies investigating the role of cGAS–STING signaling in cancer identified its role vis-à-vis type I IFN response (Diamond et al., 2011; Fuertes et al., 2011). Shortly after the discovery that the cGAS–STING pathway is the main mediator of the type I IFN response to cytosolic dsDNA, STING became an attractive anticancer drug target, which led to the development of STING agonists. Activation of STING in the tumor microenvironment (TME) was shown to impart a potent antitumor effect. In the meantime, however, multiple lines of evidence from multiple groups indicated that STING can have a pro-tumorigenic effect, in some contexts promoting metastatic progression and immune evasion.

Herein, we briefly review the current knowledge on the dichotomous functions of cGAS–STING in the TME, emphasizing that rigorous understanding of the mechanism governing this pathway in cancer is critical for the successful clinical translation of STING-directed therapies.

## The cGAS–STING signaling pathway can be triggered *in cis* and *in trans* in the TME

Despite the various mechanisms that have been reported to prohibit improper cGAS–STING activation by self-dsDNA (Chen et al., 2016b), in many pathological contexts, including cancer and autoimmune disorders, self-DNA can still activate this pathway (Figure 1). First, dsDNA fragments of genomic origins often act as the source of cytosolic dsDNA in cancer cells (Dou et al., 2017; Mackenzie et al., 2017; Bakhoum et al., 2018). This self-dsDNA can arise due to intrinsic causes, such as chromosomal instability (CIN) and nuclear herniation during cellular senescence, or as a result of genotoxic therapies. In many instances, this process involves the formation of micronuclei, where entire chromosomes or chromosome fragments become surrounded by rupture-prone nuclear envelopes. The exposure of dsDNA to the cytoplasm occurs upon micronuclear envelope rupture, which serves as a seminal event during cGAS activation.

Another potential source of self-derived cytosolic DNA is mitochondrial DNA (mtDNA). In *KRAS-LBK1* mutant lung cancer cells, dysfunctional mitochondria have been found to release mtDNA—a potent activator of cGAS—into cytosol (Kitajima et al., 2019). mtDNA can also gain access to the cytosol upon caspase activation, wherein membrane pore-forming proteins, known as gasdermin family members, permeabilize the mitochondrial membrane directly releasing the DNA (Rogers et al., 2019; Huang et al., 2020).

**Figure 2:**
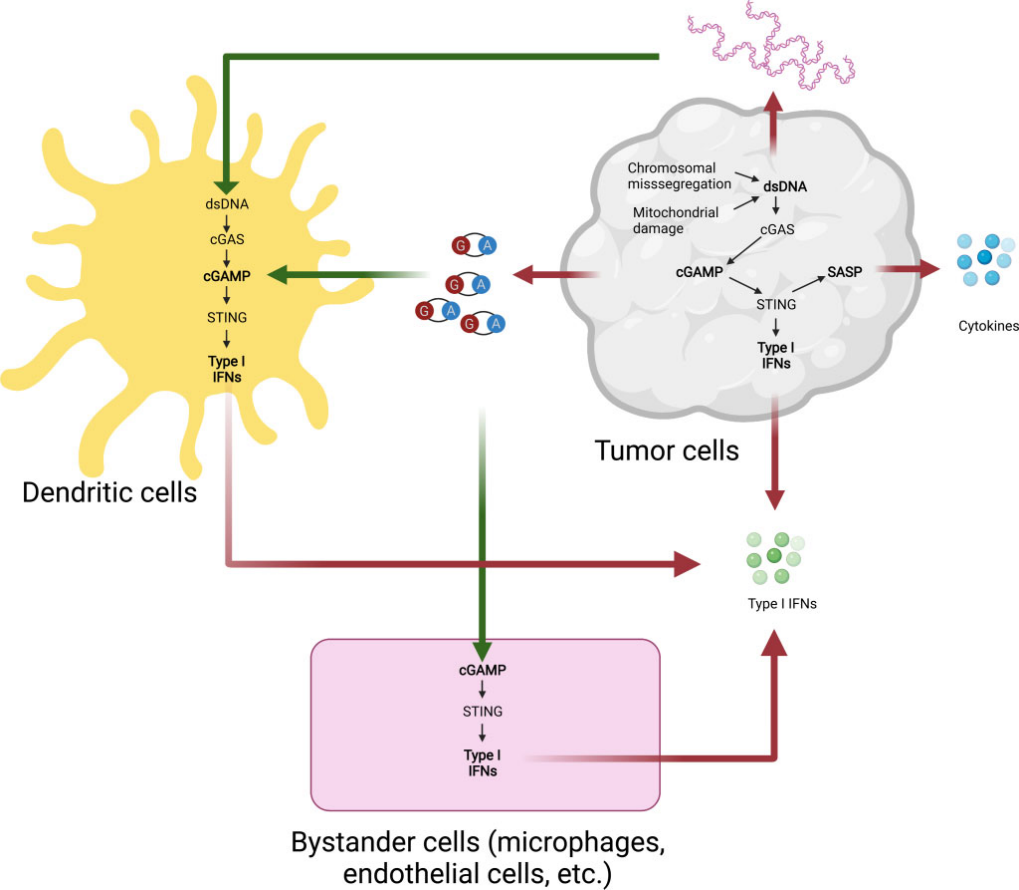
The cGAS–STING pathway is activated *in trans* and *in cis* in the TME. cGAS–STING signaling is not only activated by cytosolic DNA in tumor cells, but also can be activated by tumor cell-derived DNA or cGAMP in neighboring cells. This figure was created with BioRender.com.

The entirety of the cGAS–STING signaling cascade does not necessarily take place in the same cell (Figure 2). Cytoplasmic dsDNA itself can be transferred from one cell to another, e.g. from cancer cells to immune cells. To identify the major recipient cells of tumor cell-derived DNA, Woo et al. (2014) traced the tumor cell DNA and found that CD11c^+^ antigen-presenting cells are the major recipients where STING is activated. Furthermore, TIM3 was discovered as a negative regulator of cGAS–STING signaling in dendritic cells (DCs) through inhibiting DNA uptake (de Mingo Pulido et al., 2021). It is unclear, however, how dsDNA can access the cytosolic compartment of DCs from the extracellular space, two topologically distinct spaces. In other studies, tumor cell-derived exosomes were proposed to transfer DNA from cancer cells to DCs to initiate STING activation (Kitai et al., 2017). In line with this scenario, host cGAS, as well as STING, is critical for the full activation of this pathway, which was shown to synergize with immune checkpoint blockade in preclinical models of melanoma (Wang et al., 2017).

Shortly after the discovery of cGAMP as the intracellular second messenger, which activates STING, intercellular cGAMP transfer to bystander cells through gap junctions was demonstrated using *in vitro* systems (Ablasser et al., 2013). This was subsequently shown to occur in breast tumor models of brain metastasis, wherein cancer cells utilize gap junctions to transfer cGAMP to astrocytes (Chen et al., 2016a). cGAMP transfer does not necessitate the use of gap junctions, however. Marcus et al. (2018) reported enhanced natural killer cell-mediated antitumor activity that is dependent on the export of tumor cell-derived cGAMP in the extracellular milieu.

How do cancer cells with CIN cope with the potentially deleterious effect of cGAMP export given that they exhibit constitutive cGAS activation in response to genomic instability? Recently, Lingyin Li's group (Carozza et al., 2020a) and our group (Li et al., 2021a) independently reported that cancer cells can evade immune surveillance by hydrolyzing extracellular cGAMP in a manner dependent on the ectonucleotidase ENPP1. Interestingly, we also found that by degrading cGAMP into its basic components, AMP and GMP, ENPP1 generates the extracellular substrate, AMP, for downstream production of adenosine, a potent immune suppressor. ENPP1 was often coexpressed with another ectoenzyme, NT5E (also known as CD73), which converts AMP into adenosine. In another study, Cordova et al. (2021) discovered that monocytes, M1-polarized macrophages, NK cells, and a small population of T cells directly sense extracellular cGAMP by monitoring IRF3 phosphorylation in these cell types in the TME. The tumor-to-host cGAMP transfer model is further supported by identification of several cell type-specific plasma membrane cGAMP transporters, including SLC19A1, SLC46A2, and LRRC8 family proteins (Luteijn et al., 2019; Ritchie et al., 2019; Lahey et al., 2020; Zhou et al., 2020; Cordova et al., 2021).

## STING-mediated IRF3–type I IFN signaling and senescence-associated secretory phenotype in the TME

A substantial body of work suggests a critical role of type I IFN responses in various host cells for innate immunity and adaptive immunity (Zitvogel et al., 2015). Upon synthesis and secretion, type I IFNs bind to their receptor, the IFNα/β receptor (IFNAR), which is composed of IFNAR1 and IFNAR2 subunits that are expressed by numerous types of cells (Figure 1). Activation of IFNAR elicits the Janus kinase (JAK)–signal transducer and activator of transcription (STAT) pathway to transcriptionally induce a panel of ISGs that are involved in a broad range of downstream reactions, like proinflammatory activity, antigen presentation, apoptosis, and cellular senescence.

Cellular senescence is defined as a permanent cellular division arrest induced by DNA damage, cellular stress, or oncogene activation. More than cell cycle arrest, cellular senescence is accompanied by secretion of soluble signaling factors, proteases, and insoluble proteins, which are known as the senescence-associated secretory phenotype (SASP) (Coppé et al., 2010).

**Figure 3:**
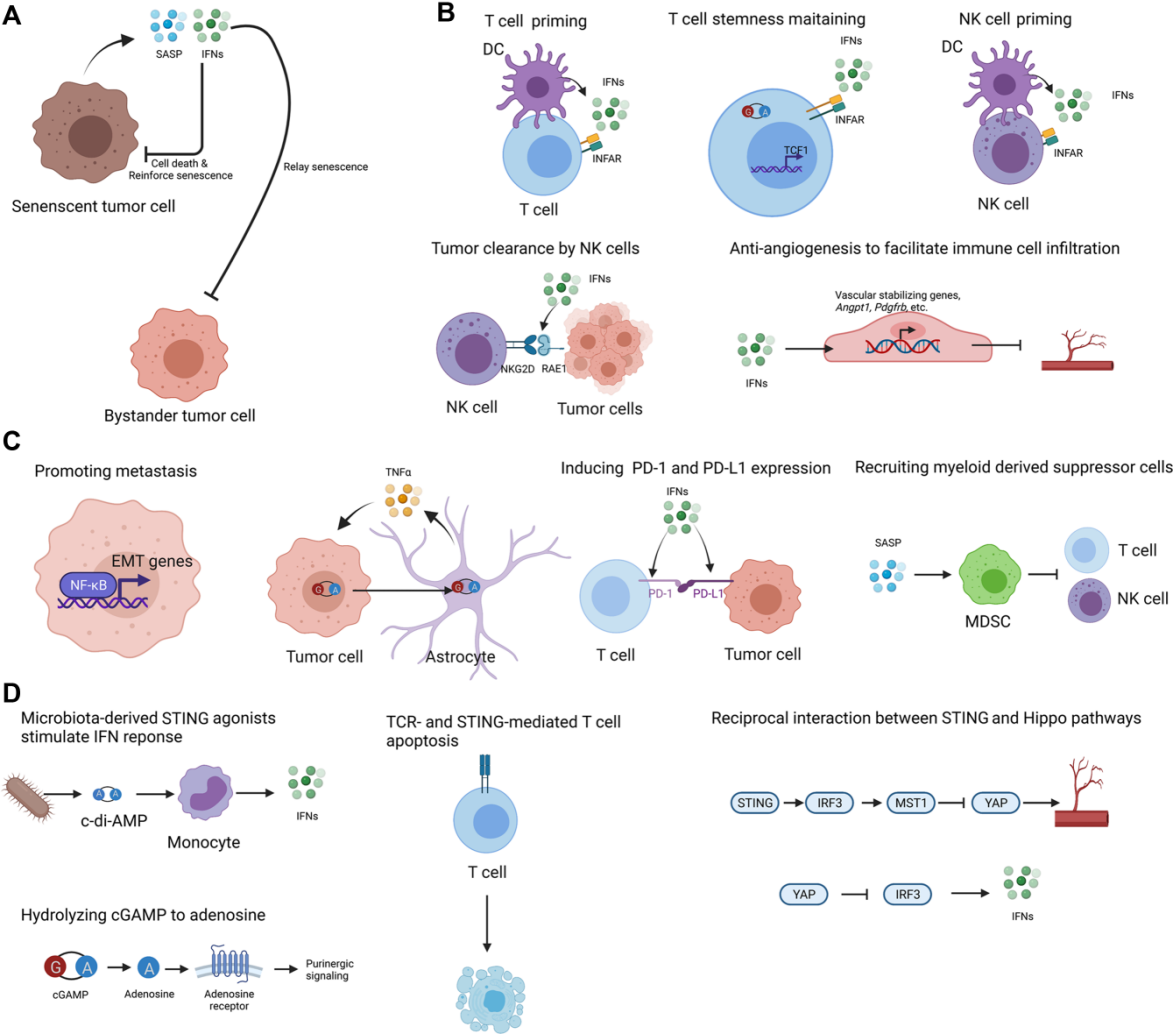
The antitumor and protumor effects of cGAS–STING activation. (**A** and **B**) STING activation can repress tumor progression directly through cell cycle arrest or cell death (**A**) by facilitating immune clearance (**B**). (**C**) Conversely, STING activation can promote tumor metastasis by inducing EMT genes in tumor cells or tumor necrosis factor alpha (TNFα) in astrocytes and can help build an immunosuppressive TME for tumor cell survival. (**D**) The cGAS–STING pathway can engage other signaling pathways in the TME modulation. This figure was created with BioRender.com.

Both type I IFNs and the SASP have been reported to have an intrinsic deleterious effect on tumor cells (Figure 3A). Katlinskaya et al. (2016) reported that type I IFNs are involved in mediating oncogene-induced cellular senescence in a murine melanoma model, suggesting that senescence-associated cell cycle arrest can be bypassed via downregulation of IFNAR. In an autocrine or paracrine manner, certain SASP factors, such as interleukin-6 (IL-6) and IL-8, are integrated in a positive feedback loop to maintain and relay a senescence state and prevent the growth of damaged cells (Acosta et al., 2008; Harlin et al., 2009). Glück et al. (2017) showed that cGAS–STING-mediated SASP factors relay cellular senescence in a type I IFN-dependent manner and mutations in the cGAS–STING pathway slow down the senescence progression. A similar observation was independently reported by Yang et al. (2017). The mechanism by which cGAS activation engages replicative senescence is still unclear. A *cGAS* mutant has more severe senescence defects than a *STING* mutant, suggesting that cGAS has both STING-dependent and STING-independent roles in cellular senescence (Glück et al. 2017; Yang et al. 2017).

The essential role of type I IFN signaling in the antitumor immune response is supported by preclinical models and analyses of clinical samples. For example, Dunn et al. (2005) showed that knocking out the gene encoding IFNAR1 in host mice, particularly in hematopoietic cells, abolished the rejection of carcinogen-induced and transplantable tumors. Positive correlations between type I IFN expression and CD8^+^ T cell infiltration in human melanoma samples were also reported (Harlin et al., 2009; Fuertes et al., 2011).

During pathogen infection, type I IFN response can be triggered by multiple innate immune responses upon danger-associated molecular pattern (DAMP) recognition by host receptors, including toll-like receptors (TLRs), P2X7R, RIG-I, and cGAS. To figure out which upstream factors are mainly responsible for type I IFN activation in the TME, Woo et al. (2014) analyzed a subset of mouse mutants with defective innate immunity-sensing signaling molecules and found that disruption of cGAS–STING, but not other receptors, reduced tumor CD8^+^ T cell infiltration and compromised host tumor control. It suggested that cytosolic DNA is the major DAMP to trigger innate immune responses in the TME, and cGAS–STING seems to be the only cytosolic DNA sensor to mediate such a function, as deletion of other intracellular DNA sensor candidates, e.g. AIM2-like receptors, did not affect the intracellular DNA-mediated IFN response (Gray et al., 2016).

Activation of type I IFN response in the TME can promote tumor clearance by the host immune system. Woo et al. (2014) reported that spontaneous rejection of methylcholanthrene-induced sarcomas in mice is cGAS-, STING-, and IRF3-dependent. A strong STING-dependent TBK1 phosphorylation can be detected in DCs isolated from tumors (Woo et al., 2014). They proposed a model in which type I IFNs can activate DCs to prime T cells to kill tumor cells (Figure 3B). Recently, Li et al. (2020) discovered that STING in T cells is critical to elicit antitumor immunity, probably via inducing type I IFN expression in a cell-autonomous manner, which in turn inhibits Akt activity and promotes the expression of genes essential for T cell stemness maintenance. The type I IFN signal can also upregulate the expression level of retinoic acid early transcript 1 (RAE1) in tumor cells. RAE1 mediates the killing of tumor cells by NK cells (Lam et al., 2014). In addition to functioning on hematopoietic cells, type I IFNs also antagonize angiogenesis by activating vascular stabilizing genes in endothelial cells (Demaria et al., 2015; Yang et al., 2019; Chelvanambi et al., 2021).

Similar to type I IFN response, senescence can stimulate immunity-mediated tumor cell clearance. Xue et al. (2007) showed that reactivating the p53 pathway in tumor cells triggered cellular senescence and inflammatory cytokine secretion, which then stimulated the innate immunity-mediated cancer-killing effect. Kang et al. (2011) established a mouse model in which oncogene-induced senescent hepatocytes were efficiently removed by the host, called ‘senescence surveillance’. Through tracing which immune cells are indispensable for senescence surveillance, Kang et al. (2011) unveiled the importance of CD4^+^ T cells in this process. Cytoplasmic chromatin fragments in senescent fibroblast cells can activate cGAS–STING signaling, which leads to NF-κB activation (Dou et al., 2017). Tumor cells that evade senescence arrest also possess the cGAS–STING–NF-κB-mediated SASP. *In vivo*, oncogene-induced senescent cells were cleared by immune systems in a cGAS–STING-dependent manner.

However, other evidence suggests that the cGAS–STING-mediated type I IFN response or SASP can have dichotomous effects inducing pro-tumorigenic functions (Boukhaled et al., 2021; Figure 3C). In an inflammation-related carcinogen model where cytosolic DNA accumulation is induced by a mutagen, STING knockout mice are more resistant to cancer induction, suggesting a carcinogenic role for chronic inflammation (Ahn et al., 2014). Various mechanisms are engaged in the tumor-promoting function of cGAS and STING. STING activation in tumor cells can induce epithelial–mesenchymal transition (EMT) gene expression to promote metastasis (Bakhoum et al., 2018). The crosstalk between tumor cells and non-tumor cells also plays vital roles. For example, the expression of the immune checkpoint mediators, PD-1 in tumor-infiltrating T cells and PD-L1 in tumor cells, can be induced by IFNα (Terawaki et al., 2011). Furthermore, radiation-induced type I IFN response can either stimulate the CD8^+^ T cell-killing effect or protect tumor cells from killing by T cells (Chen et al., 2019). During brain metastasis, cGAMP transfer from tumor cells to astrocytes has been proposed to stimulate the synthesis of IFNα and TNFα to promote metastasis (Chen et al., 2016a). Chronic inflammation during senescence might enhance tumor growth by recruiting myeloid-derived suppressor cells using the CCR2 cytokine receptor (Liang et al., 2017). Eggert et al. (2016) showed that while the CCL2–CCR2 axis helps senescent hepatocyte clearance to reduce hepatocellular carcinomas in the early stages of tumorigenesis, it imposes an immunosuppressive effect and supports tumor growth by inhibiting NK cell-mediated killing in later stages of cancer progression. Immune-suppressive M2 tumor-associated macrophages can be polarized by microparticles derived from tumor cells, and this M2 polarization depends on the cGAS–STING–TBK1–STAT6 axis in macrophages (Ma et al., 2016).

## Crosstalk between cGAS–STING signaling and other pathways in the TME modulation

Although IRF3-mediated type I IFN and NF-κB responses are the major downstream events upon cGAS–STING activation, this pathway might engage other signaling pathways to influence the TME (Figure 3D). cGAMP is the native but not the only ligand of STING *in vivo*. Recently, Lam et al. (2021) showed that proper microbiota sensitizes mice to immunotherapy by releasing c-di-AMP, a STING agonist, to stimulate type I IFN synthesis in monocytes in the TME. Furthermore, extracellular cGAMP can be not only imported by neighboring cells to activate STING in a paracrine fashion, but also hydrolyzed into AMP and GMP by a plasma membrane-resident ectonucleotide pyrophosphatase/phosphodiesterase, ENPP1 (Carozza et al., 2020a; Li et al., 2021a). AMP can be further hydrolyzed to adenosine by CD73. Adenosine is one of the purinergic signaling ligands and can stimulate a series of G-protein-coupled adenosine receptors, which are expressed by immune cells, including macrophages, DCs, neutrophils, mast cells, and lymphocytes (Ohta, 2016). Among adenosine receptors, the A_2A_ receptor is more broadly expressed by mature immune cells and has a higher affinity for adenosine, which makes it easier to activate by adenosine of physiological concentration (Fredholm et al., 2001). A_2A_ receptor activation leads to the adenylate cyclase–cAMP–protein kinase A (PKA) canonical pathway. One target of the PKA pathway is cyclic AMP-responsive element-binding protein, whose activation inhibits NF-κB.

cGAS–STING activation can trigger immune cell death in a context-dependent manner. For example, Wu et al. (2019, 2020) reported that STING activation in T cells leads to cell apoptosis in the presence of T-cell receptor signal. Interestingly, the cell death signal is independent of the IFN function of STING, as STING-S365A mutation, which disrupts IRF3 activation, cannot block cell death (Wu et al., 2020). By comparing tumor-infiltrating T cells with WT STING and STING-S365A, Wu et al. (2020) found a similar level of cell death in WT as in STING-S365A, suggesting that such IRF3-independent cell death exists *in vivo* as well.

The cGAS–STING pathway has been shown to crosstalk with the Hippo pathway, which integrates various extracellular and intracellular upstream stimuli, including cell adhesion, mechanical cues, and G-protein-coupled receptor activation. When the Hippo pathway is activated, phosphorylation of Yes-associated protein 65/transcriptional coactivator with PDZ-binding domain (YAP/TAZ) retains them in the cytosol to be degraded. When the Hippo pathway is off, unphosphorylated YAP1/TAZ enters the nuclear, functions with the transcription factor TEAD, and then activates the genes that control cell proliferation and cell fate and are thus closely related to cancer (Pan, 2010; Varelas, 2014; Calses et al., 2019). The Hippo–YAP pathway has reciprocal interaction with innate immunity pathways, including cGAS–STING signaling. For example, in endothelial cells, cGAS–STING–IRF3 directly upregulates MST1, which subsequently inhibits YAP1/TAZ and then angiogenesis (Yuan et al., 2017). The activated Hippo pathway inhibits the activation of type I IFN in a transcription-independent manner, where YAP/TAZ directly binds to TBK1 and inhibits its phosphorylation (Zhang et al., 2017).

## Perspective

Collectively, it has become clear that the role of the cGAS–STING pathway in cancer is context-dependent, which can be attributed to several factors. First, the consequences of pathway activation in cancer cells and host or immune cells can be distinct. Second, several components of the pathway can cross cellular boundaries, including the cell-to-cell transfer of DNA, cGAMP, inflammatory cytokines, and beyond. Hydrolysis of extracellular cGAMP leads to the generation of adenosine, which might have different effects depending on the receptor engaged and the cell type expressing such a receptor.

Although current research mainly focuses on models in which chromosomally unstable cancer cell-derived cGAMP is exported to bystander cells, it is interesting to test whether cGAMP can be exported by non-tumor cells and imported into tumor cells vice versa. By importing cGAMP from the TME, tumor cells that have a lower level of CIN or those with less cGAS expression can still activate the pro-tumor signaling downstream of STING, e.g. the noncanonical NF-κB pathway. This hypothesis raises the interesting possibility that senescent or virus-infected cells might contribute to tumor progression by fueling with extracellular cGAMP.

Second, cGAS–STING signaling is not a linear cascade. For example, cGAS has a STING-independent function and can be recruited to chromatin during mitosis in a cGAMP-unrelated manner, promoting cellular senescence and DNA recombination repair (Yang et al., 2017; Liu et al., 2018; Zhong et al., 2020; Li et al., 2021c). Similarly, STING can be activated by ATM and IFI16 independently of cGAS (Dunphy et al., 2018). In addition, downstream of the cGAS–cGAMP–STING cascade, multiple pathways can be differentially activated, including type I IFN, NF-κΒ, and autophagy. The cellular factors that govern the respective pathway activation under various contexts remain to be elucidated.

Given the important role of cGAS–STING in anticancer immunity, STING agonists have been developed to treat cancer. The initial generation of STING agonists are synthetic derivatives of cyclic dinucleotides (CDNs). In mouse models, direct intratumoral injection of synthetic CDNs leads to antitumor response (Corrales et al., 2015; Sivick et al., 2018). To overcome the poor stability of CDNs, researchers have successfully identified non-CDN STING agonists, including amidobenzimidazole, MSA-2, and SR-717, which can be administrated systemically rather than intratumorally (Ramanjulu et al., 2018; Chin et al., 2020; Pan et al., 2020). While many encouraging results have emerged from preclinical mouse models, early-stage clinical trials are yet to show a convincing efficacy signal for STING agonists (Motedayen Aval et al., 2020), highlighting the need to unveil the complex regulation of this pathway.

We anticipate that experimental tools and platforms that robustly detect the activation of cGAS–STING signaling *in vivo* will not only help us understand the mechanisms that regulate this pathway in a context-dependent manner, but also help us predict the overall consequence of applying STING agonists in the TME. Separation-of-function STING mutants are invaluable reagents to address this complex biology. For example, mice with STING-S365A mutation allow researchers to investigate the IFN-independent function of STING in different immune cell populations (Wu et al., 2020; Yum et al., 2021). Novel drug delivery technology will also make it possible to deliver STING agonists to certain cell populations to specifically elicit anti-immune response without activating STING where it might aid tumor progression (Wang et al., 2020; Li et al., 2021b).

Although developing STING agonists is still the major direction when speaking of targeting the cGAS–STING pathway in cancer treatment, in some patient populations, inhibition of chronic inflammation arising from STING signaling might be a more sensible approach given the role of STING in promoting metastatic progression (Bakhoum et al., 2018). Additional targets in this domain might involve blocking the transfer of cGAMP between cells or its hydrolysis in the extracellular domain through the development of ENPP1 inhibitors (Carozza et al., 2020a, b). In summary, while this pathway holds potential therapeutic promise, a deeper understanding of the biology and the development of reliable biomarkers will be needed for successful translation into the clinic.
